# Habitat Isolation Effects on Personality in a Ground Beetle, *Carabus convexus* Fabricius, 1775

**DOI:** 10.3390/insects17040356

**Published:** 2026-03-24

**Authors:** Tibor Magura, Szabolcs Mizser, Roland Horváth, Mária Tóth, Ferenc Sándor Kozma, Vanda Éva Abriha-Molnár, Bianka Sipos, Anada Takár, Gábor L. Lövei

**Affiliations:** 1 Department of Ecology, Faculty of Science and Technology, University of Debrecen, Egyetem Sq. 1, H-4032 Debrecen, Hungary; mizser.szabolcs@science.unideb.hu (S.M.); horvath.roland@science.unideb.hu (R.H.); toth.maria@science.unideb.hu (M.T.); molnarvandaeva@science.unideb.hu (V.É.A.-M.); sipos.bianka@science.unideb.hu (B.S.); 2 HUN-REN–UD Anthropocene Ecology Research Group, Egyetem Sq. 1, H-4032 Debrecen, Hungary; kozmafs@gmail.com (F.S.K.); gabor.lovei@agro.au.dk (G.L.L.); 3 Count István Tisza Foundation for the University of Debrecen, Egyetem Sq. 1, H-4032 Debrecen, Hungary; 4 Juhász-Nagy Pál Doctoral School of Biology and Environmental Sciences, University of Debrecen, Egyetem Sq. 1, H-4032 Debrecen, Hungary; anadabarbara@gmail.com; 5 Department of Agroecology, Aarhus University, Flakkebjerg Research Centre, DK-4200 Slagelse, Denmark

**Keywords:** behavior, carabid, invertebrate, novel environment test, emergence test, activity, exploration, boldness, repeatability

## Abstract

Urbanization is a major and rapidly expanding form of land-use change worldwide and is one of the main drivers of the decline in arthropod diversity. Within urban matrices, remnants of natural or semi-natural habitats serve as important refuges for native organisms. However, these urban fragments are typically small, isolated, and strongly affected by various forms of disturbance. Therefore, connectivity among urban remnant patches may enhance population persistence and resilience. Increased tendencies to explore novel environments, tolerate human disturbance, and exploit unpredictable resources can be advantageous in urban environments. Accordingly, in this study of a flightless ground beetle species, we hypothesized that individuals from urban habitats—especially from isolated ones—would be bolder and more exploratory than their rural conspecifics, that sexes would differ in behavior, and that these behaviors would be temporally consistent, indicating animal personality. Activity-, exploration-, and boldness-related behavioral traits were significantly repeatable, providing evidence for animal personality, particularly in females and rural beetles. Contrary to our hypothesis, no behavioral differences were detected between rural and urban individuals. Furthermore, no significant sex-dependent differences in behavior were observed. Overall, our findings highlight the importance of species-specific traits and ecological context in shaping behavioral variation.

## Simple Summary

Urbanization is expanding rapidly around the world and is a major cause of the decline in insect diversity. In cities, small patches of natural habitat, such as forest fragments, can act as refuges for native species, but these areas are often isolated and exposed to strong human disturbance. Understanding how animals behave in such environments can help us predict which species are able to survive in cities. In this study, we examined the behavior of a flightless ground beetle living in rural and urban habitats. We focused on traits such as activity, willingness to explore new surroundings, and boldness (how quickly an individual leaves a safe shelter). We expected beetles from urban, especially isolated, habitats to be bolder and more exploratory than rural individuals and to differ between males and females. We found that individual beetles showed consistent behavioral differences over time, indicating stable “personalities”, particularly in females and rural beetles. However, beetles from urban and rural habitats behaved similarly, and males and females did not differ significantly. These results show that behavioral responses to urbanization depend strongly on species-specific traits and ecological context, highlighting the need for species-focused approaches in urban conservation.

## 1. Introduction

Urbanization is a global and rapidly expanding form of land-use change, creating a particular kind of human-modified environment [1]. Urbanization involves the spatial increase of human density, and its subsequent replacement of natural and semi-natural habitats with built infrastructure, impervious surfaces [2,3]. Cities/settlements typically experience elevated temperatures (the urban heat island effect [4]), reduced infiltration and increased runoff [5], and higher levels of chemical [2], light [6], and noise [7] pollution.

As a result, the original habitat becomes fragmented into “habitat islands” within urban matrices. These can serve as important refuges for native flora and fauna and maintaining ecosystem functions [7]. However, such habitat remnants are usually small, spatially isolated, and strongly influenced by edge effects, causing increased exposure to disturbance, invasive species, and altered abiotic conditions [8]. Populations inhabiting these remnant patches often occur at lower densities and face an elevated risk of local extinction [9]. Connections to surrounding rural landscapes and/or other remnant habitat patches can facilitate dispersal, gene flow, and recolonization, thereby enhancing population persistence [10,11]. In contrast, isolated urban habitat patches may function as demographic sinks, where populations persist only temporarily without sufficient immigration [12,13].

The environmental changes associated with urbanization can strongly influence how individuals interact with their surroundings and may favor specific traits that enhance performance under urban conditions [7,14]. Sets of correlated behavioral traits, described as behavioral syndromes, may further constrain or facilitate responses to urban environments by linking multiple behaviors along common axes such as boldness, activity, or risk-taking [15,16].

Urban-dwelling individuals of mammals [17], birds [18], reptiles [19], and also invertebrates [20], are more willing to explore novel environments, tolerate human disturbance, and exploit unpredictable or anthropogenic resources [21]. Despite the growing body of evidence supporting increased boldness and exploration in urban populations, the strength and direction of rural–urban behavioral differences vary considerably by context, among species, and even sexes [22].

Our study aimed to test whether activity-, exploration-, and boldness-related behavioral traits differ in the ground beetle *Carabus convexus* originating from rural habitats, or urban forest patches connected to or isolated from rural areas. Specifically, we hypothesized that (H1) measures quantifying behavioral traits are repeatable across trials, indicating the presence of consistent individual differences in behavior (animal personality). Further, (H2) individuals from isolated urban habitats are bolder and more exploratory than rural conspecifics, and (H3) differences exist in behavioral traits and their repeatability between males and females, because of their different reproduction-related strategies. We expected males to be more exploratory and bolder than females.

We found that all measures of behavioral traits were repeatable across trials for females but not for males, providing clear evidence for the presence of animal personality. However, neither habitat type nor sex had a significant effect on activity, exploration, or boldness.

## 2. Materials and Methods

### 2.1. Study Area and Sampling Design

The research was conducted in forested habitats located both within and near the administrative boundaries of Debrecen (47°32′ N, 21°38′ E), Hungary’s second largest city, which had 201,704 residents in 2024 and covers 461.7 km^2^. The city lies adjacent to a 1092 ha large mature (>120 years old) lowland oak forest (*Convallario*–*Quercetum roboris*), protected since 1992. Urban expansion dating back to the early 20th century created several isolated urban remnants of the formerly continuous forest (Figure S1). This situation offers a framework for investigating urbanization-related ecological changes under comparable mesoclimatic conditions, geomorphology, soil characteristics, forest type, age, and land-use history.

To assess the ecological consequences of habitat fragmentation by urbanization, we selected six forest sites, including two rural and four urban ones, all at least 250 m apart. This inter-patch distance ensured the independence of samples [23] and, because of known carabid movement patterns, also spatially distinct ground beetle assemblages [24,25]. The selected sites were similar in size and sufficiently large (all above 4.5 ha) to support self-sustaining arthropod assemblages [26]. Urban sites were embedded in landscapes with a markedly higher proportion of urban land use (built-up and impervious surfaces, urban parks, ponds) compared to rural ones (62.97 ± 6.21% vs. 1.17 ± 0.18% within a 1000 m buffer, respectively; Table S1). The urban sites differed in their connection to rural habitats (Figure S1), with two of them being connected to (connected urban sites hereafter), while the other two were isolated from rural habitats (isolated urban sites hereafter). Isolated urban sites were separated from the rural habitats by built-up and impervious surfaces or two-lane asphalt roads. Asphalt-paved surfaces like roads function as effective barriers for flightless ground beetles, as individuals rarely cross them [27,28].

Beetles were sampled from mid-March to the end of May 2024, using live, unbaited pitfall traps. At each sampling site, 15 traps were deployed, resulting in a total of 90 traps (6 sites × 15 traps). The traps were placed randomly, with a minimum distance of 10 m between them and a distance of at least 50 m from the forest edge to minimize potential edge effects [29]. Traps consisted of plastic containers (170 mm in length, 110 mm in width, and 105 mm in depth) partially filled with shredded leaf litter to provide refuge and reduce intraguild predation. Each trap was covered with a 20 × 20 cm fiberboard lid to protect against rain. Traps were checked twice per week. Captured beetles were transported to the laboratory, identified to species level, sexed, and their live body mass was measured (two repeats, precision 0.1 mg). After weighing, beetles were housed individually in Petri dishes (90 mm diameter) with moistened filter paper with access to water but no food.

### 2.2. Study Species

*Carabus convexus* Fabricius, 1775, is a medium-sized, wingless, widely distributed Eurasian forest specialist ground beetle [30]. In Central Europe, adult activity typically starts in late March, with egg laying from mid-April onward [31]. Newly emerged (teneral) individuals can be found from late July, and adults of this new generation overwinter in November. Collectively, these traits—strong habitat affiliation, medium body size, and limited dispersal ability—make *C. convexus* vulnerable to the ecological impacts of urbanization [32,33,34]. In *C. convexus*, there is a behavioral difference by season. During the reproduction period, both sexes are more exploratory than beetles after the reproduction [35]. For this reason, we decided to concentrate on the reproduction period that is crucial for population persistence. This has the additional advantage of avoiding collecting too many individuals from small urban populations. During the sampling period, 123 *C. convexus* individuals (65 females and 58 males) were captured. We collected 83 individuals from rural sites, 33 beetles from connected urban sites, and 7 individuals from isolated urban sites. All individuals had minimally worn mandibles, suggesting that they had overwintered and were entering their first reproductive season.

### 2.3. Behavioral Assays

Following transport from the field, during warm periods in a cool box, individuals were allowed a 2 h acclimatization and recovery period prior to behavioral assays. Locomotor activity, exploration, and boldness were first quantified in a novel environment assay (also called open-field test) [36,37,38]. Subsequently, exploratory and boldness/anxiety-related behaviors were measured in an emergence test [39,40]. To prevent observer bias, experimenters were blind to the origin of the individual.

The novel environment consisted of a white plastic arena (364 × 230 mm) with the floor subdivided into 35 equal-sized squares [41]. At the start, a randomly chosen beetle was placed in the central square and covered with a Petri dish (55 mm diameter). Once the individual remained motionless for at least five seconds, the cover was carefully removed without physical contact, and the beetle’s movements were video-recorded for 90 s [20] using a GoPro HERO6 camera (CHDHX-601-FW, GoPro, Inc., San Mateo, CA, USA). Video recordings were analyzed with BugTracker (version 0.2 [42]) and Windows Movie Maker (version 8.0.7.5), and the following behavioral measures were quantified: (1) the total number of squares visited (hereafter referred to as “no. squares visited”), representing overall locomotor activity [37] or exploratory behavior [20]; (2) the number of non-peripheral squares (non-adjacent to the arena wall) visited (“no. inner squares visited”), used as a measure of exploration [37] or boldness, as higher frequency of entry into the center of the arena (centrophilia) indicate bolder behavior [40]; (3) the time until the beetle started to move (“latency to move”), interpreted as a measure of exploration or boldness [43].

During the emergence test, a randomly selected beetle was placed in a semitransparent brown plastic vial (length: 6 cm, diameter: 2.5 cm), which was sealed and positioned horizontally. After 1 min of acclimatization, the cap of the vial was removed, enabling the beetle to leave. We measured the time needed for the beetle to (4) emerge from the shelter (“latency to emerge”). The test was stopped when the head of the beetle was completely out of the vial or after a maximum of 5 min. If the beetle did not leave the vial, it received a score of 300 s. This measure quantifies the exploratory behavior or boldness [39,40,43].

The repeatability of the above measures was assessed by performing each assay twice, separated by 24 h [37,44]. Limiting the assessment to two trials minimized the risk of habituation; increasing the number of trials does not enhance repeatability [45].

### 2.4. Statistical Analyses

All statistical analyses were conducted in the R environment (version 4.5.2 [46]). Body mass may affect arthropod behavior [40]; thus, it was included in the analyses. The effects of habitat type, sex, body mass, and their interactions on behavioral measures were examined using generalized linear mixed models (GLMMs). Prior to model fitting, the most appropriate probability distributions were determined using the *car* (version 3.1-3 [47]) and MASS (version 7.3-65 [48]) packages. Count-type behavioral measures (no. squares visited, no. inner squares visited) were modeled using a Poisson distribution, whereas time-related ones (latency to move, latency to emerge) were analyzed using a lognormal error distribution with the *glmmTMB* package (version 1.1.14 [49]). The hierarchical structure of the experimental design was accounted for by including sampling sites nested within habitat type as a random effect. Trials, experimenter identity, and individual beetle identity (ID) were incorporated as additional random factors. Model-estimated marginal mean differences between habitat types were calculated using the *emmeans* package (version 2.0.2 [50]). To examine whether the results changed when urban areas were treated together, we ran an additional GLMM where connected and isolated urban habitats were not separated.

To assess whether individual beetles ranked similarly across behavioral measures, the Kendall’s coefficient of concordance was calculated using the *DescTools* package (version 0.99.60 [51]) separately for the two trials. Consistency between repeated trials was quantified by estimating adjusted repeatability from GLMMs that included individual identity as a random term, using the *rptR* package (version 0.9.23 [52]). These analyses were performed first for all individuals combined and subsequently for females and males, as well as for beetles from rural, connected, and isolated urban sites separately. Finally, potential associations among behavioral measures were explored by calculating Spearman’s rank correlation coefficients between behavioral measures for each trial separately.

## 3. Results

Individual beetles were consistently ranked across behavioral measures in both trials (Kendall’s coefficient of concordance: trial 1, W = 0.220, χ^2^ = 81.020, df = 3, *p* < 0.0001; trial 2, W = 0.030, χ^2^ = 11.146, df = 3, *p* = 0.011), providing evidence for the existence of structured behavioral patterns.

When data from female and male beetles were pooled, all measured behavioral measures showed significant repeatability (Table 1). In contrast, significant repeatability across behavioral variables was observed only in females (Table 1). Among males, repeatability was detected only for the number of squares visited, whereas latency to move and latency to emerge were marginally significant, and the number of inner squares visited did not show significant repeatability (Table 1). When data were analyzed separately for beetles collected from rural, connected, and isolated urban sites, significant repeatability across behavioral variables was observed only in rural beetles (Table 2).

Neither habitat type, sex, nor body mass, nor their interactions had a significant effect on any of the studied behavioral measures (Table 3). The effect of habitat type on the latency to move was negligible (χ^2^ = 0.414, *p* = 0.813; a model-estimated marginal mean difference between rural and connected urban sites of −0.441 [95% CI: −1.320, 0.440], and between rural and isolated urban sites of −1.936 [95% CI: −5.710, 1.830]). The effect of habitat type on the number of squares visited was also negligible (χ^2^ = 0.854, *p* = 0.652; a model-estimated marginal mean difference between rural and connected urban sites of −0.077 [95% CI: −0.621, 0.776], and between rural and isolated urban sites of 0.603 [95% CI: −2.630, 3.837]). The effect of habitat type on the number of inner squares visited was also negligible (χ^2^ = 1.650, *p* = 0.438; model-estimated marginal mean difference of 0.029 [95% CI: −0.536, 0.594] between rural and connected urban sites, and −0.028 [95% CI: −2.795, 2.740] between rural and isolated urban ones). The effect of habitat type on the latency to emerge was also negligible (χ^2^ = 1.900, *p* = 0.387; model-estimated marginal mean difference of −0.220 [95% CI: −0.491, 0.052] between rural and connected urban sites, and −0.231 [95% CI: −1.257, 0.795] between rural and isolated urban ones). The same result was obtained when connected urban and isolated urban habitats were combined and treated as an urban habitat type (Table S2).

**Figure 1:**
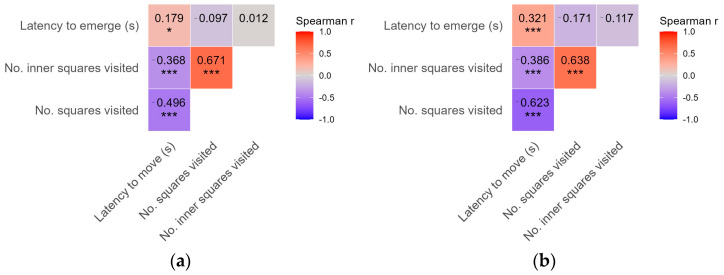
Correlation matrix of the studied behavioral measures using Spearman’s rank correlation coefficient for the first (**a**) and second trial (**b**). Colors indicate the strength and direction of correlations (blue = negative; red = positive). Only the upper triangle is shown. Numbers represent Spearman’s correlation coefficients, and asterisks indicate significance levels (* *p* < 0.05, *** *p* < 0.001). Behavioral measures were recorded in a novel environment (latency to move, No. squares visited, No. inner squares visited) and an emergence behavior test (latency to emerge).

Several of the behavioral measures studied were significantly correlated with each other, and in some cases significant correlations were also detected between measures quantified in the novel environment and emergence tests. The strongest positive correlation occurred between the number of squares visited and the number of inner squares visited (Figure 1). In contrast, both of these were significantly negatively correlated with latency to move. A significant positive correlation was also found between latency to emerge from shelter and latency to move in the novel environment test (Figure 1).

## 4. Discussion

### 4.1. Limitations of the Dataset

Our results are based on a single species in a single year. Including more species and temporal replication would strengthen the possibility of generalization. Nonetheless, we believe that information concerning a forest specialist carabid is still useful and potentially relevant for other ground beetles of similar ecological requirements.

Despite intensive, standardized sampling with identical sampling effort across all three habitat types, *C. convexus* abundance differed markedly among habitats, with very few individuals captured in the isolated urban fragments. Consequently, our results should be interpreted with caution. The pronounced decline along the rural–connected urban–isolated urban habitat gradient is consistent with previous findings demonstrating the sensitivity of *C. convexus* to urbanization [32,33,34]. Earlier studies conducted in the same region further support this pattern, indicating that only small populations persist in urban forest fragments [53], particularly in those isolated by paved roads and other impervious surfaces [54,55].

The small sample size from the isolated urban sites and, therefore, the imbalance in sample size among habitat types compromised the statistical power of our models, as GLMMs are sensitive to such imbalances. All the above factors require caution when interpreting our results.

### 4.2. Behavior Repeatability and Time Interval Between Trials

A previous meta-analysis [45] showed that repeatability is higher when repeated trials are closer in time. Therefore, the relatively short interval between repeated behavioral trials in our study may have influenced the repeatability estimates. However, nearly two-thirds (64.95%) of the experiments included in the above-mentioned meta-analysis concerned vertebrate species, and a rather coarse temporal categorization was used (less than or more than one year) [45]. Because behavioral repeatability may differ between vertebrate and invertebrate species—and even among different invertebrate taxa [56]—and because invertebrates often have shorter development than a year, a new summary of data on repeatability of beetle behavior is warranted.

We collected published repeatability estimates of behavioral measures in beetles using the novel environment and emergence behavioral tests [20,36,41,57,58,59,60,61,62] (Table S3)—both of which were applied in our study. Repeatability estimates assessed in a novel environment did not change significantly as a function of the time interval between repeated trials, either when the sexes were analyzed together or when females were analyzed separately (Table S4). In contrast, when males were analyzed separately, repeatability estimates increased significantly with increasing time intervals between trials. Based on these results, we recommend that in studies of beetle behavior using the novel environment test, the interval between repeated observations should not exceed 5 days. This relatively short interval would ensure that the studied beetles remain in comparable states (e.g., hunger, body mass, physiological condition, etc.), thereby minimizing the confusing effects of environmental and state-dependent variation on behavioral repeatability [45].

### 4.3. Animal Personality

In behavioral ecology, behavioral traits refer to measurable aspects of behavior, including but not limited to activity levels, boldness, exploratory tendency, risk-taking, and aggression. Such traits often exhibit considerable variation among individuals and are typically assessed using standardized protocols under controlled laboratory or field conditions [15]. When individual differences in a given behavioral trait remain consistent over time and/or across different situations, this consistency is interpreted as evidence for animal personality [15,56,63].

Based on the correlations among behavioral measures quantified in the novel environment and emergence tests, the strong and significant correlation between the number of squares visited and the number of inner squares visited in the novel environment arena indicates, in line with previous conclusions [20,37,64], that these two variables reliably quantify activity and exploratory behavior of ground beetles. In contrast, the significant, moderate positive correlation between latency to move in the novel environment test and latency to emerge from the shelter in the emergence test suggests that these measures may serve as accurate indicators of boldness and exploration in insects [39,40,43]. The strength of correlation was higher between latency to move and latency to emerge in the second trial than in the first one. This leads to a methodological recommendation that one should allow a one-day acclimatization for the lab-transported beetles, in order to minimize the effect of stress linked to capture and transport. The moderate, significant negative correlation between latency to move and the number of both the total and the inner squares visited in the novel environment test is due solely to methodological limitations, as beetles that start movement later in the arena certainly visit fewer squares.

In our study, all activity-, exploration-, and boldness-related behavioral traits quantified in novel environment and emergence behavioral tests were significantly repeatable over time, either when sexes were analyzed together or when females were assessed separately, proving evidence for animal personality in *C. convexus*. Repeatability estimates of behavioral measures were lower when male and female beetles were analyzed together than when females were analyzed separately, suggesting substantial variability in the repeatability of male behavior. Indeed, in males, only activity- and exploration-related behavior—quantified as the number of squares visited in the novel environment test—showed significant repeatability. Thus, our hypothesis was only partially confirmed, as we did not confirm the presence of personality in male *C. convexus* beetles.

Male behavior has often been considered more repeatable than female behavior, a pattern commonly attributed to hormonal influences such as testosterone that may increase behavioral predictability in males [45,65], as well as to the role of sexually selected behavioral traits in mate choice [45]. Nevertheless, according to a recent meta-analysis, behaviors unrelated to mate preference exhibit higher repeatability in females than in males, thereby making the prevailing assumption of greater behavioral repeatability in males equivocal [45]. Similarly, we found higher repeatability in females, while in males, most behavioral measures were not significantly repeatable. Higher repeatability of activity-, exploration-, and boldness-related behaviors in female than male beetles may reflect sex-specific differences in life-history strategies and behavioral plasticity. In females, these behaviors are closely linked to energetic demands and reproductive investment, potentially favoring stable, individual-specific behavioral strategies. In contrast, male behavior may be more context-dependent and responsive to mating opportunities, resulting in increased within-individual variation and lower repeatability [56]. This pattern is consistent with growing evidence from invertebrate taxa indicating that sex differences in behavioral repeatability do not necessarily conform to expectations derived from studies on vertebrates [66]. However, previous findings on the exploratory behavior of ground beetles are inconsistent. Studies on *Carabus hortensis* Linnaeus, 1758 [61] and *Nebria brevicollis* (Fabricius, 1792) [20] reported higher repeatability in males than females, whereas a study on *Pterostichus oblongopunctatus* (Fabricius, 1787) [20], similarly to our results, found higher repeatability in females than males. Consequently, future studies investigating sex-specific differences in behavioral repeatability are essential for drawing general conclusions.

We showed that activity-, exploration-, and boldness-related behavioral measures were significantly repeatable over time in rural but not in urban *C. convexus*. Consistent individual differences in behavior are expected to occur when environmental conditions are relatively predictable and consistent behavior is favored by selection [67,68]. Rural habitats are typically characterized by reasonably stable environmental conditions; therefore, maintaining a fixed behavioral strategy may be advantageous, resulting in behavioral repeatability. In contrast, urban habitats with frequent disturbances are less predictable, favoring behavioral flexibility over consistency [69].

### 4.4. Urbanization and Behavior

Human-induced disturbances (such as traffic, impervious surfaces, and diverse pollutants), together with the environmental modifications associated with urbanization, generate novel and often challenging environments for urban survivors. Certain behavioral traits may facilitate successful adaptation to such environments. Research on such traits has predominantly focused on vertebrates—most commonly birds and mammals—and has demonstrated that urban individuals tend to exhibit higher levels of boldness and exploratory behavior than their rural conspecifics [70,71,72,73]. Similar patterns have been reported in several arthropod taxa, including grasshoppers [74], ants [75], and spiders [76].

In contrast, our results indicated no significant differences in activity, exploration, or boldness between rural and urban adults of *C. convexus*. Similarly, a previous study, assessing the activity- and exploration-related behavioral measures in a novel environment, found no significant differences in these measures between *C. convexus* individuals from forested rural and isolated urban forest fragments [41]. To date, only one other study has investigated urbanization-related differences in activity and exploratory behavior of ground beetles in the city of Hamburg, Germany [20]. This study reported no significant differences between beetles collected from forest patches with low versus high levels of urbanization in three of the four species studied (*Abax parallelepipedus* (Piller & Mitterpacher, 1783), *N. brevicollis* and *P. oblongopunctatus*). Individuals of the fourth species, *Carabus nemoralis* O.F. Müller, 1764, showed significantly higher activity and exploratory behavior at highly urbanized sites than elsewhere. However, this effect was detected based on a single field test, preventing any assessment of behavioral consistency or repeatability. Moreover, the less urbanized forest patches were still located within the city and thus represented suburban rather than rural habitats [20]. Consequently, the reported behavioral differences should be interpreted with caution.

The absence of behavioral differences among rural, connected, and isolated urban populations of *C. convexus* may be explained by several mechanisms, which are not necessarily mutually exclusive. Due to the limited dispersal capacity of this flightless species, urban populations probably represent remnants persisting after urban expansion. In this context, urbanization may primarily act as a strong environmental filter, allowing only pre-adapted individuals to survive [77], without necessarily promoting further behavioral divergence. Shifts in other behavioral traits related to microhabitat use, thermal tolerance, or daily activity patterns may play a role in adaptation to urban environments [78]. In addition, structurally similar microhabitats in urban forest fragments to those in rural ones may reduce the perceived novelty [79]. Finally, the relatively short time since urbanization, combined with a multi-year generation time, may have limited the emergence of detectable evolutionary differentiation in behavior [80].

### 4.5. Sex-Dependent Behavior

Sex-related variation in behavioral traits is common across a wide range of animal taxa and is often associated with differences in reproductive strategies and life-history trade-offs between males and females [66]. In many species, males typically display more exploration and risk-taking, a pattern frequently attributed to sexual selection favoring active mate searching. By contrast, females often show more cautious behavior, which may reflect stronger selection pressure on survival and future reproductive investment [56,66].

Contrary to our hypothesis, we found no significant differences between female and male *C. convexus* in activity, exploration, and boldness. In the studied habitats, both sexes appear to experience similar ecological constraints and selective pressures, which likely minimized sex-specific behavioral differences. Similarly, a meta-analysis involving 37 invertebrate species, including four ground beetles, reported no significant sex-dependent differences in activity, exploration, or boldness [81]. Previous studies examining the activity and exploratory behavior in *C. hortensis* and *C. nemoralis* found that females were significantly less active and exploratory, visiting fewer squares within the arena [20,61]. In contrast, in an earlier novel environment test, female *C. convexus* showed significantly higher activity and exploration than males [41]. These contradictory findings highlight that sex-related behavioral differences are strongly trait-, taxon-, and context-dependent. The strong influence of these factors is further supported by the high heterogeneity in effect size estimates reported in a meta-analysis examining sex differences in animal personality [81].

## 5. Conclusions

In this study, we investigated the behavioral repeatability, personality traits, and effects of urbanization and sex on the behavior of the flightless ground beetle *C. convexus*. Behavioral analyses demonstrated significant repeatability in activity-, exploration-, and boldness-related traits, providing evidence for animal personality, particularly in rural females. Repeatability estimates support the presence of meaningful among-individual behavioral variation. In contrast, behavioral consistency in males was weaker, suggesting sex-specific differences in behavioral plasticity. Contrary to our hypothesis, no behavioral differences were detected between rural and urban individuals, indicating that urbanization has not led to measurable divergence in the traits studied. This pattern likely reflects the limited dispersal capacity of *C. convexus*, the persistence of remnant urban populations, and similar microhabitat conditions across forested sites. Furthermore, no significant sex-dependent differences in behavior were observed, suggesting that both sexes experience comparable ecological constraints. Overall, our findings highlight the importance of species-specific traits, ecological context, and sex in shaping behavioral variation and emphasize the need for further studies on invertebrate behavior across environmental gradients.

## Figures and Tables

**Table 1:** Adjusted repeatability (R) of the behavioral measures of adult *Carabus convexus*, as well as females and males collected from rural and differently isolated urban habitats. Behavioral measures were recorded in a novel environment (latency to move, no. squares visited, no. inner squares visited) and an emergence behavior test (latency to emerge). Values in bold denote significant repeatability (*p* < 0.05), while those in italics denote marginally significant repeatability (*p* < 0.1).

Response Variable	Repeatability, R [95% CI] *
***All individuals* (*n* = 123)**	
Latency to move	**0.297 [0.117, 0.432]**
No. squares visited	**0.292 [0.071, 0.427]**
No. inner squares visited	**0.191 [0, 0.327]**
Latency to emerge	**0.229 [0.052, 0.384]**
***Females* (*n* = 65)**	
Latency to move	**0.330 [0.091, 0.521]**
No. squares visited	**0.310 [0.042, 0.508]**
No. inner squares visited	**0.343 [0.001, 0.522]**
Latency to emerge	**0.304 [0.052, 0.489]**
***Males* (*n* = 58)**	
Latency to move	*0.204 [0, 0.426]*
No. squares visited	**0.271 [0, 0.45]**
No. inner squares visited	0 [0, 0.187]
Latency to emerge	*0.178 [0, 0.422]*

* confidence intervals (CIs) were calculated using 1000 bootstraps.

**Table 2:** Adjusted repeatability (R) of the behavioral measures of adult *Carabus convexus* individuals collected from rural and differently isolated urban habitats. Behavioral measures were recorded in a novel environment (latency to move, no. squares visited, no. inner squares visited) and an emergence behavior test (latency to emerge). Values in bold denote significant repeatability (*p* < 0.05).

Response Variable	Repeatability, R [95% CI] *
***Individuals from isolated urban sites* (*n* = 7)**	
Latency to move	0.426 [0, 0.901]
No. squares visited	0 [0, 0.5]
No. inner squares visited	0 [0, 0.626]
Latency to emerge	0 [0, 0.558]
***Individuals from connected urban sites* (*n* = 33)**	
Latency to move	0 [0, 0.296]
No. squares visited	0.066 [0, 0.338]
No. inner squares visited	0.094 [0, 0.378]
Latency to emerge	0.112 [0, 0.421]
***Individuals from rural sites* (*n* = 83)**	
Latency to move	**0.428 [0.232, 0.57]**
No. squares visited	**0.420 [0.176, 0.563]**
No. inner squares visited	**0.231 [0, 0.424]**
Latency to emerge	**0.229 [0.008, 0.428]**

* Confidence intervals (CI) were calculated using 1000 bootstraps.

**Table 3:** Analysis of deviance table for the fitted generalized linear mixed models on the behavioral measures of *Carabus convexus* adults collected from rural and differently isolated urban habitats. Behavioral measures were recorded in a novel environment (latency to move, no. squares visited, no. inner squares visited) and an emergence behavior test (latency to emerge).

Response Variable	Explanatory Variable	χ^2^	d.f.	*p*
***Latency to move***				
	Habitat	0.414	2	0.813
	Sex	0.000	1	0.997
	Body mass	0.681	1	0.409
	Habitat × Sex	0.592	2	0.744
	Habitat × Body mass	0.223	2	0.894
	Sex × Body mass	0.032	1	0.858
	Habitat × Sex × Body mass	0.556	2	0.757
***No. squares visited***				
	Habitat	0.854	2	0.652
	Sex	0.977	1	0.323
	Body mass	0.276	1	0.599
	Habitat × Sex	0.124	2	0.940
	Habitat × Body mass	0.594	2	0.743
	Sex × Body mass	1.070	1	0.301
	Habitat × Sex × Body mass	0.050	2	0.975
***No. inner squares visited***				
	Habitat	1.650	2	0.438
	Sex	0.009	1	0.923
	Body mass	0.029	1	0.865
	Habitat × Sex	0.132	2	0.936
	Habitat × Body mass	1.531	2	0.465
	Sex × Body mass	0.003	1	0.959
	Habitat × Sex × Body mass	0.339	2	0.844
***Latency to emerge***				
	Habitat	1.900	2	0.387
	Sex	1.776	1	0.183
	Body mass	0.713	1	0.399
	Habitat × Sex	2.484	2	0.289
	Habitat × Body mass	1.862	2	0.394
	Sex × Body mass	2.025	1	0.155
	Habitat × Sex × Body mass	2.518	2	0.284

## Data Availability

The data presented in this study are openly available in Mendeley repository at doi: 10.17632/wwyxbzr63t.1, https://data.mendeley.com/datasets/wwyxbzr63t/1 (accessed on 23 February 2026).

## Supplementary Materials

The following supporting information can be downloaded at: https://www.mdpi.com/article/10.3390/insects17040356/s1, Figure S1. Schematic map showing the mature lowland oak forest north of the city of Debrecen and its urban remnant patches (“Great forest”) as well as the land use types (“Impervious surface”—built-up and impervious surfaces; “Urban forest”—managed, middle-aged forest stands with mainly native canopy-forming species; “Urban pond”—artificially created standing water; “Urban park”—intensively managed, more open urban forested parks with native and/or non-native species in the canopy layer, or intensively managed parks dominated by open habitat patches with scattered native and/or non-native trees). ”R1” and “R2”—rural sampling sites; “Uc1” and “Uc2”—urban sampling sites connected to rural forest; “Ui1” and “Ui2”—urban sampling sites isolated from rural forest; Figure S2. Schematic image of (A) the novel environment arena, with inner squares marked in gray; (B) the plastic vial for testing the emergence behavior; Figure S3. Model estimates (and 95% CI) for the fitted generalized linear mixed models on the behavioral measures of *Carabus convexus* individuals collected from rural and connected (“urbancon”) and isolated urban (“urbaniso”) sites. Behavioral measures were recorded in a novel environment (A: latency to move, B: no. squares visited, and C: no. inner squares visited) and an emergence behavior test (D: latency to emerge); Table S1. Area of the sampling sites, as well as the proportion of modified habitats (urban park, urban pond, built-up and impervious surfaces) within a 1000 m buffer around the sampling sites. See Figure S1 for location of the sampling sites and the land-use map; Table S2. Analysis of deviance table for the fitted generalized linear mixed models on the behavioral measures of *Carabus convexus* individuals collected from rural and urban habitats (connected and isolated urban sites were treated together). Behavioral measures were recorded in a novel environment (latency to move, no. squares visited, no. inner squares visited) and an emergence behavior test (latency to emerge); Table S3. Interval between trials and repeatability estimates for behavioral measures assessed in novel environment assays for various beetle species; Table S4. Summary of the fitted generalized linear mixed models on the effect of the time interval (in days) on the repeatability estimates for behavioral measures assessed in novel environment tests using published data on beetles extracted during a systematic review (see Table S3). Models were fitted for all individuals and then separately for females and males. In the models, repeatability estimates were used as the response variable, with a Gaussian error distribution, while the time interval between repeated trials (in days) was set as the explanatory variable. Study identity, species identity, and behavioral measures were included as random effects. *p* values in bold denote significant effects (*p* < 0.05).
