# Supplementary material for: Habitat Isolation Effects on Personality in a Ground Beetle, Carabus convexus Fabricius, 1775
Source: Insects. 2026 Mar 24;17(4):356. doi: 10.3390/insects17040356 (PMC13116161; doi:10.3390/insects17040356)
Supplement: Supplementary file 1 [file insects-17-00356-s001.zip › insects-4195321-supplementary.pdf]

Supplementary material to the article

Habitat Isolation Effects on Personality in a Ground Beetle, *Carabus convexus* Fabricius, 1775

by Tibor Magura, Szabolcs Mizser, Roland Horváth, Mária Tóth, Ferenc S. Kozma, Vanda É. Abriha-Molnár, Bianka Sipos, Anada Takár, and Gábor L. Lövei

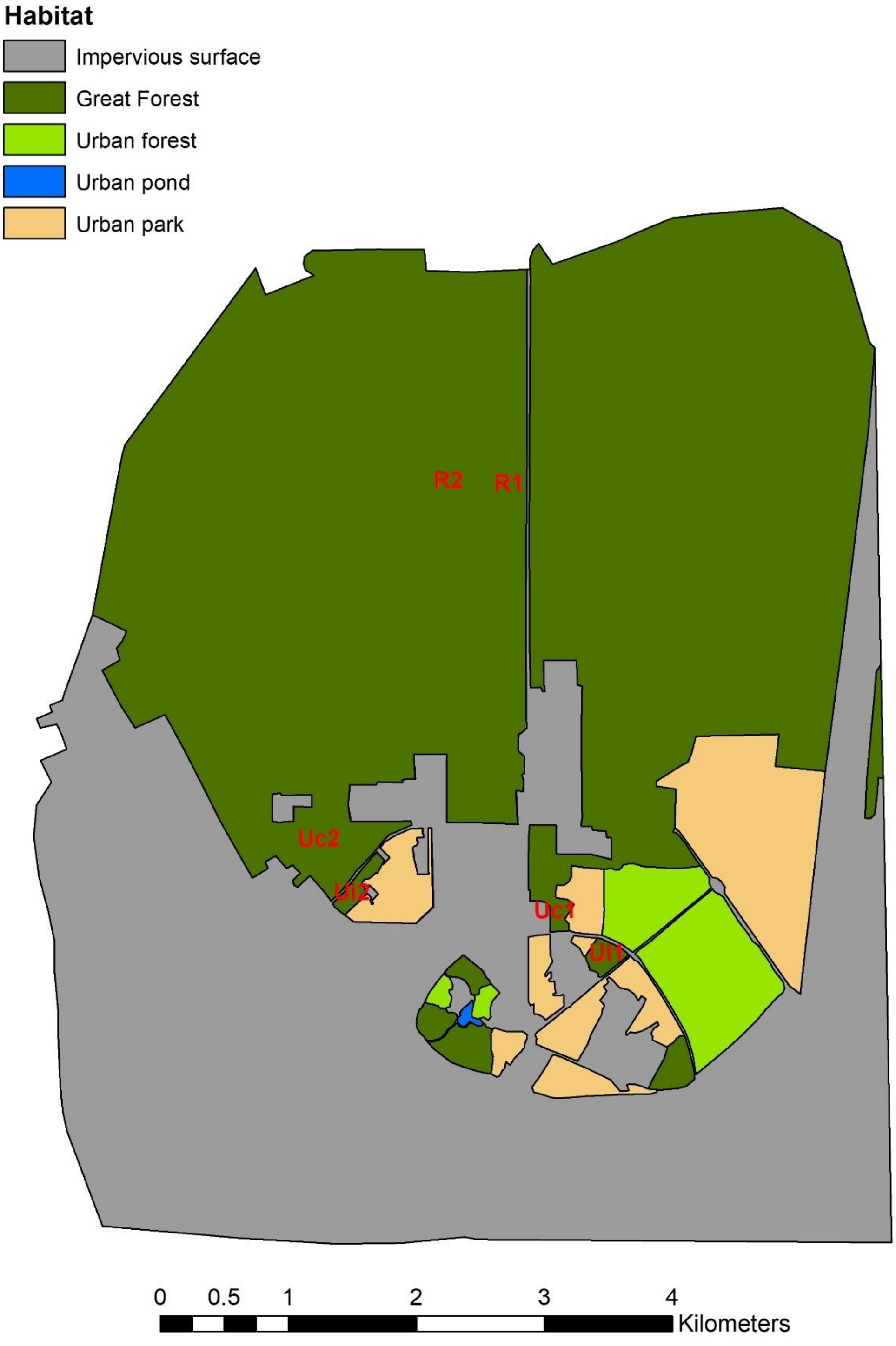

Figure S1. Schematic map showing the mature lowland oak forest north of the city of Debrecen and its urban remnant patches ("Great forest") as well as the land use types ("Impervious surface" – built-up and impervious surfaces; "Urban forest" – managed, middle-aged forest stands with mainly native canopy-forming species; "Urban pond" – artificially created standing water; "Urban park" – intensively managed, more open urban forested parks with native and/or non-native species in the canopy layer, or intensively managed parks dominated by open habitat patches with scattered native and/or non-native trees)."R1" and "R2" – rural sampling sites; "Uc1" and "Uc2" – urban sampling sites connected to rural forest; "Ui1" and "Ui2" – urban sampling sites isolated from rural forest.

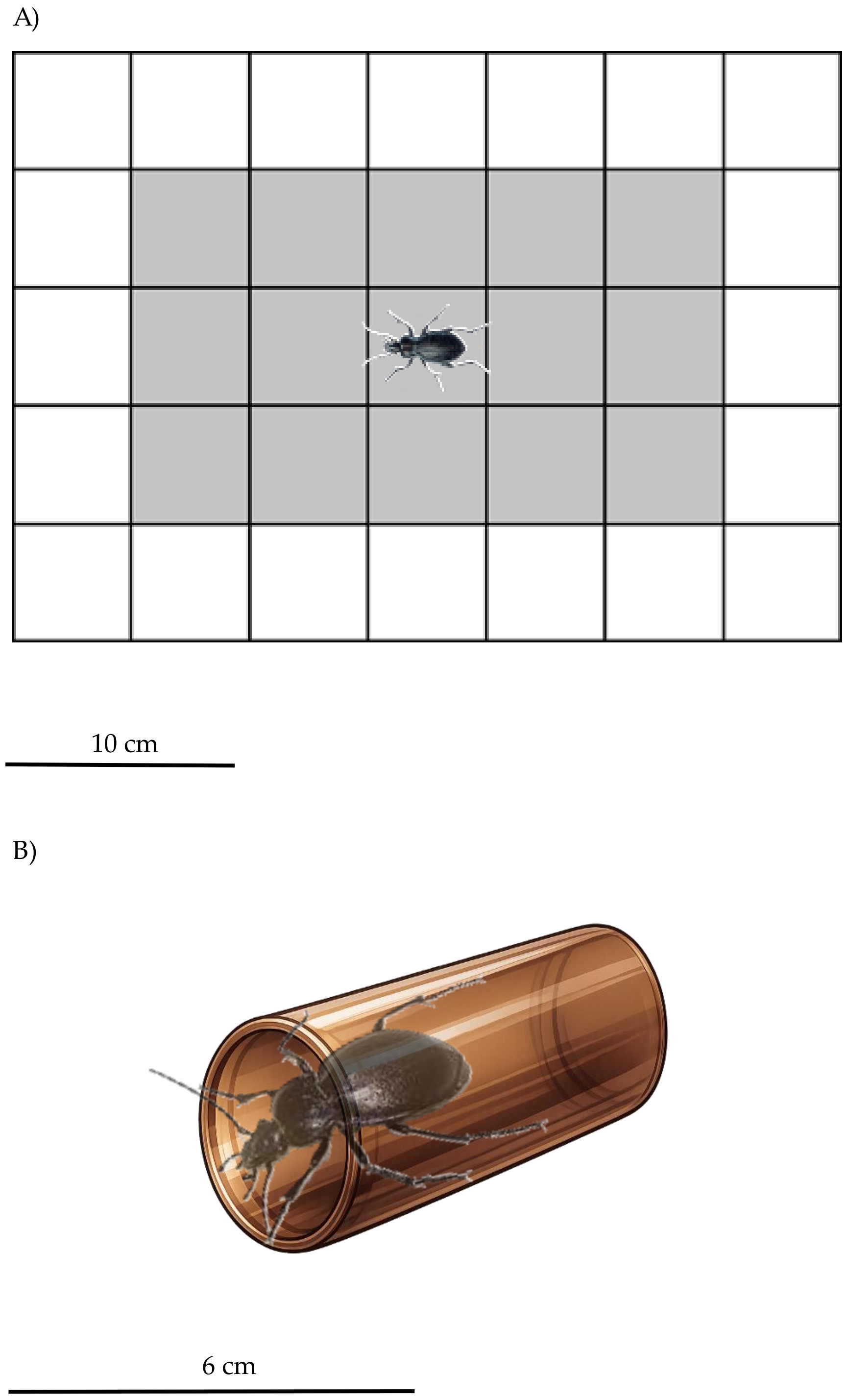

Figure S2. Schematic image of (A) the novel environment arena, with inner squares marked by grey, and (B) the plastic vial for testing the emergence behavior.

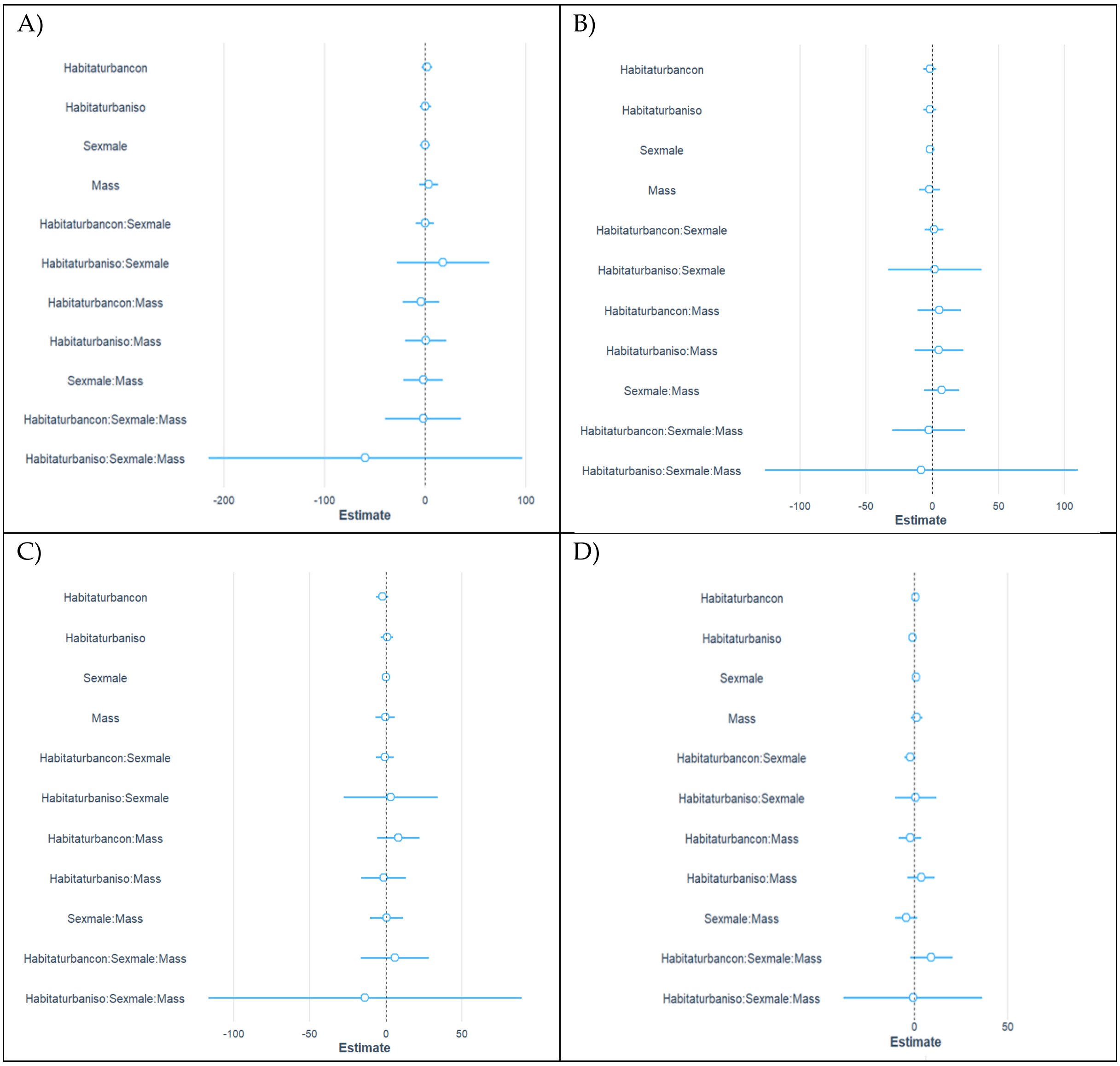

Figure S3. Model estimates (and 95% CI) for the fitted generalized linear mixed models on the behavioral measures of *Carabus convexus* individuals collected from rural and connected (“urbancon”) and isolated urban (“urbaniso”) sites. Behavioral measures were recorded in a novel environment (A: latency to move, B: no. squares visited, C: no. inner squares visited) and an emergence behavior test (D: latency to emerge).

Table S1. Area of the sampling sites, as well as the proportion of modified habitats (urban park, urban pond, built up and impervious surfaces) within a 1000m buffer around the sampling sites. See also Figure S1 for location of the sampling sites and the land-use map.

	**Area (ha)**	**Proportion (%) of modified habitats within a 1000m buffer**
**Rural site 1 (R1)**	6.490	1.298
**Rural site 2 (R2)**	6.002	1.046
**Urban connected site 1 (Uc1)**	4.751	63.479
**Urban connected site 2 (Uc2)**	6.625	59.843
**Urban isolated site 1 (Ui1)**	5.943	57.142
**Urban isolated site 2 (Ui2)**	6.186	71.429

Table S2. Analysis of deviance table for the fitted generalized linear mixed models on the behavioral measures of *Carabus convexus* individuals collected from rural and urban habitats (connected and isolated urban sites were treated together). Behavioral measures were recorded in a novel environment (latency to move, no. squares visited, no. inner squares visited) and an emergence behavior test (latency to emerge).

**Response variable**	**Explanatory variable**	χ^2	**d.f.**	p
Latency to move				
	Habitat	0.263	1	0.608
	Sex	0.000	1	0.995
	Body mass	0.683	1	0.408
	Habitat × Sex	0.084	1	0.772
	Habitat × Body mass	0.066	1	0.798
	Sex × Body mass	0.032	1	0.857
	Habitat × Sex × Body mass	0.061	1	0.805
No. squares visited				
	Habitat	1.007	1	0.316
	Sex	0.946	1	0.331
	Body mass	0.277	1	0.599
	Habitat × Sex	1.100	1	0.294
	Habitat × Body mass	0.790	1	0.374
	Sex × Body mass	1.049	1	0.306
	Habitat × Sex × Body mass	0.885	1	0.347
No. inner squares visited				
	Habitat	0.152	1	0.696
	Sex	0.009	1	0.924
	Body mass	0.028	1	0.868
	Habitat × Sex	0.004	1	0.951
	Habitat × Body mass	0.175	1	0.676
	Sex × Body mass	0.002	1	0.961
	Habitat × Sex × Body mass	0.001	1	0.983
Latency to emerge				
	Habitat	0.007	1	0.935
	Sex	1.770	1	0.183
	Body mass	0.704	1	0.402
	Habitat × Sex	1.808	1	0.179
	Habitat × Body mass	0.031	1	0.860
	Sex × Body mass	2.016	1	0.156
	Habitat × Sex × Body mass	2.351	1	0.125

Table S3. Interval between trials and repeatability estimates for behavioral measures assessed in novel environment assays for various beetle species.

Studied species*	Studied family	Studied behavioral measure	Sex	Day(s) between trials	Repeatability estimate (*R*)	Study
Carabus convexus	Carabidae	square visit	not distinguished	1	0.518	Magura et al. 2021 [1]
Carabus convexus	Carabidae	distance	not distinguished	1	0.474	Magura et al. 2021 [1]
Carabus convexus	Carabidae	motion time	not distinguished	1	0.431	Magura et al. 2021 [1]
Carabus convexus	Carabidae	inner square visit	not distinguished	1	0.313	Magura et al. 2021 [1]
Carabus convexus	Carabidae	time to wall	not distinguished	1	0.402	Magura et al. 2021 [1]
Carabus hortensis	Carabidae	square visit	not distinguished	14	0.367	Yarwood et al. 2021 [2]
Carabus hortensis	Carabidae	square visit	females	14	0.247	Yarwood et al. 2021 [2]
Carabus hortensis	Carabidae	square visit	males	14	0.484	Yarwood et al. 2021 [2]
Carabus problematicus	Carabidae	distance trackball	not distinguished	7	0.35	Roald-Arbøl et al. 2025 [3]
Nebria brevicollis	Carabidae	square visit	not distinguished	3	0.66	Harris et al. 2020 [4]
Nebria brevicollis	Carabidae	square visit	not distinguished	5	0.277	Schuett et al. 2018 [5]
Nebria brevicollis	Carabidae	square visit	females	5	0.249	Schuett et al. 2018 [5]
Nebria brevicollis	Carabidae	square visit	males	5	0.321	Schuett et al. 2018 [5]
Pterostichus oblongopunctatus	Carabidae	square visit	not distinguished	5	0.272	Schuett et al. 2018 [5]
Pterostichus oblongopunctatus	Carabidae	square visit	females	5	0.271	Schuett et al. 2018 [5]
Pterostichus oblongopunctatus	Carabidae	square visit	males	5	0.247	Schuett et al. 2018 [5]
Onthophagus taurus	Scarabaeidae	time outside	females	2	0.124	Zhang et al. 2025 [6]
Onthophagus taurus	Scarabaeidae	time outside	males	2	0.117	Zhang et al. 2025 [6]
Onthophagus taurus	Scarabaeidae	time to object	females	2	0.111	Zhang et al. 2025 [6]
Onthophagus taurus	Scarabaeidae	time to object	males	2	0.113	Zhang et al. 2025 [6]
Nicrophorus vespilloides	Silphidae	distance	males	3	0.29	Prokop et al. 2025 [7]
Nicrophorus vespilloides	Silphidae	distance	females	3	0.48	Prokop et al. 2025 [7]
Nicrophorus vespilloides	Silphidae	time inner	males	3	0.05	Prokop et al. 2025 [7]
Nicrophorus vespilloides	Silphidae	time inner	females	3	0.13	Prokop et al. 2025 [7]
Abemus chloropterus	Staphylinidae	square visit	not distinguished	1	0.183	Magura et al. 2022 [8]
Abemus chloropterus	Staphylinidae	inner square visit	not distinguished	1	0	Magura et al. 2022 [8]
Abemus chloropterus	Staphylinidae	time to wall	not distinguished	1	0.191	Magura et al. 2022 [8]

Ocypus nitens	Staphylinidae	square visit	not distinguished	1	0.332	Magura et al. 2022 [8]
Ocypus nitens	Staphylinidae	inner square visit	not distinguished	1	0.067	Magura et al. 2022 [8]
Ocypus nitens	Staphylinidae	time to wall	not distinguished	1	0	Magura et al. 2022 [8]
Platydracus fulvipes	Staphylinidae	square visit	not distinguished	1	0.446	Magura et al. 2022 [8]
Platydracus fulvipes	Staphylinidae	inner square visit	not distinguished	1	0.143	Magura et al. 2022 [8]
Platydracus fulvipes	Staphylinidae	time to wall	not distinguished	1	0.247	Magura et al. 2022 [8]
Tribolium castaneum	Tenebrionidae	distance	males	3	0.422	Li et al. 2025 [9]
Tribolium castaneum	Tenebrionidae	emergence time	males	3	0.393	Li et al. 2025 [9]
Tribolium castaneum	Tenebrionidae	time to object	males	3	0.119	Li et al. 2025 [9]

* We identified 39 studies by a literature search on Web of Science for the period 1975–2015, using the following search terms: beetle* (Topic) and behavio* (Topic) and repeatability OR repeatable OR consistent* (Topic) and personality (Topic). To be included, a study had to report data on repeatability estimate for a behavioral measure, as well as time interval between the repeated trials. The listed nine studies met this criterion, yielding a total of 36 independent estimates.

References

1. Magura, T.; Mizser, S.; Horváth, R.; Nagy, D.D.; Tóth, M.; Csicsek, R.; Lövei, G.L. Are there personality differences between rural vs. urban-living individuals of a specialist ground beetle, Carabus convexus? *Insects* **2021**, *12*, 646, doi:10.3390/insects12070646.
2. Yarwood, E.; Drees, C.; Niven, J.E.; Schuett, W. Sex-specific covariance between metabolic rate, behaviour and morphology in the ground beetle Carabus hortensis. *PeerJ* **2021**, *9*, e12455, doi:10.7717/peerj.12455.
3. Roald-Arbøl, M.; Yarwood, E.; David Fernandes, A.S.; Moubarak, E.M.; Drees, C.; Niven, J.E.; Schuett, W. Using a low-cost trackball system to assess repeatability of movement in small animals. *Ecol. Evol.* **2025**, *15*, e72535, doi:10.1002/ece3.72535.
4. Harris, C.; Liedtke, J.; Drees, C.; Schuett, W. Exploratory behaviour is not related to associative learning ability in the carabid beetle Nebria brevicollis. *Behav. Processes* **2020**, *180*, 104224, doi:10.1016/j.beproc.2020.104224.
5. Schuett, W.; Delfs, B.; Haller, R.; Kruber, S.; Roolfs, S.; Timm, D.; Willmann, M.; C, D. Ground beetles in city forests: does urbanization predict a personality trait? *PeerJ* **2018**, *6*, e4360, doi:10.7717/peerj.4360.
6. Zhang, L.; Baas, K.B.; Biesot, L.; de Boer, A.; Nugteren, A.; Székely, T.; Komdeur, J. Multidimensional behavioral consistency and syndromes in the dung beetle Onthophagus taurus: adult sex ratio has no effect on personality expression. *Behav. Ecol. Sociobiol.* **2025**, *79*, 72, doi:10.1007/s00265-025-03613-4.
7. Prokop, P.; Balcečík, J.; Masarovič, R.; Provazník, Z. Examining personalities and behavioural syndromes in the burying beetle, Nicrophorus vespilloides Herbst, 1783. *Ecol. Evol.* **2025**, *15*, e71718, doi:10.1002/ece3.71718.
8. Magura, T.; Horváth, R.; Mizser, S.; Tóth, M.; Nagy, D.D.; Csicsek, R.; Balla, E.; Lövei, G.L. Urban individuals of three rove beetle species are not more exploratory or risk-taking than rural conspecifics. *Insects* **2022**, *13*, 757, doi:10.3390/insects13080757.
9. Li, R.; Yang, C.; Lou, Y.; Lü, J.; Lyu, N. Animal personalities influence male mating behavior and reproductive fitness. *Behav. Ecol.* **2025**, *36*, arae094, doi:10.1093/beheco/arae094.

Table S4. Summary of the fitted generalized linear mixed models on the effect of the time interval (in days) on the repeatability estimates for behavioral measures assessed in novel environment tests using published data on beetles extracted during a systematic review (see Table S3). Models were fitted for all individuals, and then separately for females and males. In the models, repeatability estimates were used as the response variable with a Gaussian error distribution, while the time interval between repeated trials (in days) was set as the explanatory variable. Study identity, species identity, and behavioral measure were included as random effects. *p* values in bold denote significant ($p < 0.05$) effects.

Time interval between trials (explanatory variable)	Estimate ± S.E.	χ^2	*p*
All individuals	-0.006 ± 0.016	0.147	0.702
Females	-0.001 ± 0.002	0.419	0.517
Males	0.0228 ± 0.009	6.867	**0.009**
